# Author Correction: Frontotemporal coordination predicts working memory performance and its local neural signatures

**DOI:** 10.1038/s41467-021-23915-1

**Published:** 2021-06-15

**Authors:** Ehsan Rezayat, Mohammad-Reza A. Dehaqani, Kelsey Clark, Zahra Bahmani, Tirin Moore, Behrad Noudoost

**Affiliations:** 1 School of Cognitive Sciences, Institute for Research in Fundamental Sciences (IPM), Tehran, Iran; 2 Cognitive Systems Laboratory, Control and Intelligent Processing Center of Excellence (CIPCE), School of Electrical and Computer Engineering, College of Engineering, University of Tehran, Tehran, Iran; 3 Department of Ophthalmology and Visual Sciences, University of Utah, Salt Lake City, UT USA; 4 Department of Biomedical Engineering, Tarbiat Modares University, Tehran, Iran; 5 Department of Neurobiology Stanford University, Stanford, CA USA; 6 Howard Hughes Medical Institute, Stanford University, Stanford, CA USA

**Keywords:** Working memory, Sensory processing

Correction to: *Nature Communications* 10.1038/s41467-021-21151-1, published online 17 February 2021.

The original version of this article contained an error in Fig. 1, in which for the two left-hand plots depicted in Fig. 1c, the time labels (and associated dotted lines) on the *x*-axis were incorrectly shifted to the right.

The correct version of Fig. 1 is:
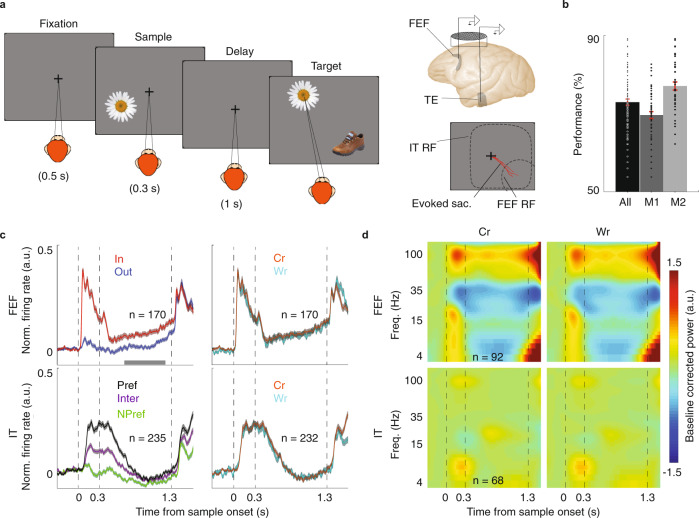


which replaces the previous incorrect version:
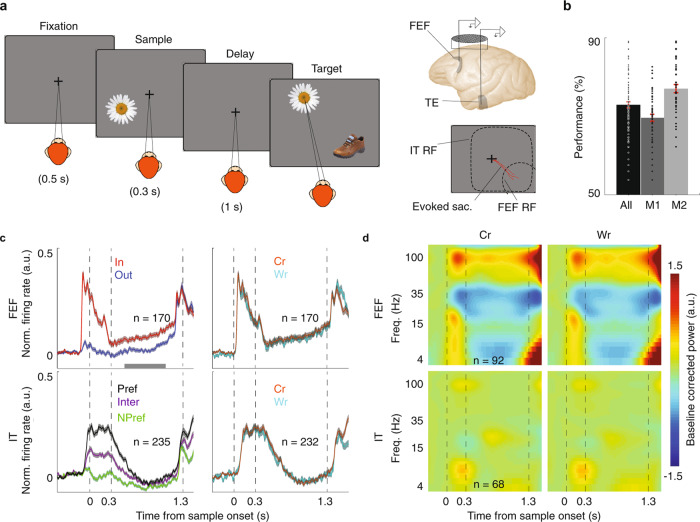


This has been corrected in both the PDF and HTML versions of the Article.

