# Supplementary material for: Frontotemporal coordination predicts working memory performance and its local neural signatures
Source: Nat Commun. 2021 Feb 17;12:1103. doi: 10.1038/s41467-021-21151-1 (PMC7889930; doi:10.1038/s41467-021-21151-1)
Supplement: Supplementary file 1 — Supplementary Information [file 41467_2021_21151_MOESM1_ESM.pdf]

Supplementary Results

Additional analysis of behavioral performance:

Reaction times (RT) were slower and variability in saccade landing points (SL_{std}) was lower on correct trials (Fig. S1a-b; $\Delta RT = 3.552 \pm 0.583$, $p < 10^{-6}$, $\Delta SL_{std} = -0.199 \pm 0.034$, $p < 10^{-6}$, n = 86). Performance as a function of sample and target eccentricity is shown in figure S1c; M1 performed the task at a greater average eccentricity and had lower average performance. The locations of estimated FEF RF centers (and sample positions) in the horizontal and vertical dimensions are shown in figure S1d. Eye position and microsaccades have been shown to modulate neural responses[1–3]; average eye position and microsaccade rates are plotted in figure S1e,f. Although the eye position was biased toward the sample location (In vs. Out, $\Delta Xpos = 0.266 \pm 0.040$, $p < 10^{-7}$, $\Delta Ypos = -0.021 \pm 0.013$, p=0.193, n = 86) there was no difference in either eye position or microsaccade rate for correct vs. wrong trials ($\Delta Xpos = 0.024 \pm 0.012$, p =0.190, $\Delta Ypos = 0.013 \pm 0.009$, p= 0.256, $\Delta MicrosaccadeRate = -0.075 \pm 0.062$, p = 0.306, n = 86).

Spike sorting isolation quality:

We cross validated the spike sorting quality using an SVM Classifier. Figure S2a shows the average classifier performance in categorizing the spike waveforms of simultaneously recorded neurons across all sessions. The overall high performance of the classifier indicates that the spikes of each sorted cluster are well isolated from each other cluster ($\Delta Perf_{IT} = 98.80\% \pm 2.50$, n = 301 pairs, $p < 10^{-50}$, $\Delta Perf_{FEF} = 98.67\% \pm 4.018$, n = 105 pairs, $p < 10^{-18}$ compared to chance level, i.e. 50%).

To doubly control for the possibility of oversorting, we verified results involving spiking activity using only a single randomly selected spiking unit from each recording session (Fig. S2b). For the difference in SPL for correct vs. wrong trials (Fig. 4a), we measured the distribution of the median difference in correct vs. wrong SPL for 1000 random subsamples of 79 spike-LFP pairs, each with a maximum of one unit per recording session. For > 99.5% of the subsamples the SPL for correct was greater than wrong, and the median of this distribution was significantly greater than zero ($\Delta SPL_{Cr\text{-}Wr} = 0.008 \pm 0.000$, $p < 10^{-10}$, n = 1000). For the modulation of object selectivity on High vs. Low PPL trials (Fig. 5e), we calculated the distribution of the median difference in modulation index (modulation of object discriminability for High vs. Low PPL trials) for In vs. Out, for 1000 subsamples (each with 40 units, a maximum of one unit per recording session, Fig. S2c). For 100% of the subsamples the modulation index was greater for In vs. Out trials, and the median of this distribution was significantly greater than zero ($\Delta MI_{In\text{-}Out} = 0.090 \pm 0.000$, $p < 10^{-10}$, n = 1000). Thus, the significance of the SPL and MI results did not depend on having multiple spiking units from the same electrode included in the analysis. Additionally, we tested whether neurons recorded from the same electrode showed a correlation in their SPL and MI effects. We calculated the correlation between the effect reported in figure 4a (SPL correct – wrong) for pairs of neurons recorded on the same electrode, and found no correlation (Fig. S2d; r = 0.033, p = 0.210). Similarly, for the MI effect from figure 5e (MI In-Out), there was no correlation in effect size between neurons recorded on the same electrode (r = 0.1, p = 0.110). We further compared the magnitude of these correlation values with the distribution of correlations for randomly selected pairs of neurons recorded on different electrodes (Fig. S2e-f); there was no significant difference between the correlation observed for same-electrode pairs and the median of the distribution of correlations for different-electrode pairs (correlation for same vs. different electrode pairs: SPL, p = 0.160; MI, p = 0.100). Altogether, these analyses confirm that sorting multiple spiking units from single electrode recordings did not artificially inflate the magnitude or significance of the reported effects.

Spatial selectivity of neural activity in the FEF:

During the delay period, there was an increase in FEF high gamma band power ($\Delta power = 0.226 \pm 0.060$, $p < 10^{-4}$, n = 92 sites) and a decrease in other frequency bands of FEF power compared to baseline

($\Delta power_{\theta}$ = -0.549 ± 0.033, $p < 10^{-15}$, $\Delta power_{\alpha}$ = -0.176 ± 0.029, $p < 10^{-14}$, $\Delta power_{\beta}$ = -0.445 ± 0.040, $p < 10^{-15}$, $\Delta power_{L\gamma}$ = -0.231 ± 0.028, $p < 10^{-11}$, n = 92 sites; Fig. 1d top). Power during the delay period also differed based on sample location for these bands (Table S2).

Object selectivity of neural activity in IT:

The IT population did not exhibit a significant increase in spiking activity during the delay period compared to baseline for the preferred object (ΔNFR = -0.005 ± 0.007, p = 0.465, n = 235), but did show a significant decrease in spiking activity for the non-preferred object (ΔNFR = -0.052 ± 0.006, $p < 10^{-18}$, n = 235). During the target period, IT firing rates were elevated when the animal saccaded to the preferred object (Cr vs. Wr, ΔNFR = 0.015± 0.004, $p <10^{-4}$, n =232).

In IT, there was a decrease in power in the high gamma and low gamma bands during the delay period compared to baseline ($\Delta power_{L\gamma}$ = -0.055 ± 0.012, $p < 10^{-6}$, $\Delta power_{H\gamma}$ = -0.035 ± 0.006, $p < 10^{-6}$, n = 69 sites). High gamma band IT power during the delay period differed for the preferred vs. non-preferred stimulus (Δpower = 0.011 ± 0.006, p = 0.013, n = 69 sites).

Controls for PPL analysis:

In order to control for differences in the number of trials, we repeated the PPL calculation using a trial matching procedure (Methods, Fig. S5a left), and found that in the beta band PPL was still greater for correct trials (ΔPPL = 0.494 ± 0.151, p = 0.001, n = 63).

The PPL statistics and data presented in figure 2 and the main text used a shuffling procedure to remove any effect of within-area phase locking (see Methods). Without this shuffling, there was still significantly higher beta band PPL on correct vs. wrong trials (Fig. S5a right; ΔPPL = 0.347± 0.100, p = 0.002, n = 63). PPL values, not shuffle-corrected, increased in the theta, alpha, and beta bands during the visual period vs. baseline (ΔPPL_{θ} = 1.000 ± 0.157, $p <10^{-7}$, ΔPPL_{α} = 0.737 ± 0.141, $p <10^{-4}$, ΔPPL_{β} = 0.286 ± 0.095, p = 0.008, n = 69). During the visual period, only the beta band PPL was object selective (ΔPPL = 0.404± 0.139, p = 0.004, n = 69) and theta, alpha, and beta band were location selective (ΔPPL_{θ} = 0.388 ± 0.119, p = 0.002, ΔPPL_{α} = 0.334 ± 0.116, p = 0.037, ΔPPL_{β} = 0.483 ± 0.128, p <0.001, n = 69). During the visual period there was significantly higher theta, alpha, and beta band PPL on correct vs. wrong trials (ΔPPL_{θ} = 0.391 ± 0.113, p = 0.003, ΔPPL_{α} = 0.336 ± 0.129, p = 0.017, ΔPPL_{β} = 0.453 ± 0.128, p = 0.001, n = 63).

Shuffle-corrected PPL values increased in the alpha and beta bands during the visual period vs. baseline (ΔPPL_{α} = 0.218 ± 0.084, p = 0.037, ΔPPL_{β} = 0.279 ± 0.112, p = 0.025, n = 69). During the visual period, only the beta band PPL was object selective (ΔPPL = 0.457 ± 0.172, p = 0.011, n = 69) and location selective (ΔPPL = 0.546 ± 0.158, p < 0.001, n = 69). During the visual period there was significantly higher beta band PPL on correct vs. wrong trials (Fig. S5a right; ΔPPL = 0.453 ± 0.128, p = 0.001, n = 63).

There was a correlation between the magnitude of FEF's spatial selectivity and the difference in FEF-IT beta band PPL between correct and wrong trials (r = 0.220, $p<10^{-5}$, n = 136; Fig. S6); this correlation was significant in both monkeys (Table S1).

Phase locking within areas across trials:

LFP phase has been shown to lock to stimulus onset[4], and so we measured the phase-locking value (PLV) of LFP oscillations to the sample onset across trials, within FEF and IT. Within FEF, PLV in the theta, alpha, and beta bands increased during the visual period compared to baseline (ΔPLV_{θ} = 1.556 ± 0.106, $p < 10^{-15}$, ΔPLV_{α} = 1.860 ± 0.182, $p < 10^{-15}$, ΔPLV_{β} = 1.268 ± 0.176, $p < 10^{-9}$, n = 92 sites). During the visual period, PLV differed between the In and Out conditions only in the theta band (ΔPLV = 0.534 ± 0.102, $p < 10^{-5}$, n = 92). During the delay period, PLV in the theta, alpha, and high gamma bands decreased compared to baseline (ΔPLV_{θ} = -1.405 ± 0.071, $p < 10^{-15}$, ΔPLV_{α} = -0.998 ± 0.092, p

$< 10^{-14}$, $\Delta PLV_{\alpha} = -0.112 \pm 0.049$, $p = 0.025$, $n = 92$ sites). During the delay period, PLV differed between the In and Out conditions in the theta and alpha bands ($\Delta PLV_{\theta} = -0.825 \pm 0.088$, $p < 10^{-11}$, $\Delta PLV_{\alpha} = -0.268 \pm 0.087$, $p = 0.001$, $n = 92$ sites). There was a significant difference between PLV in the theta and alpha bands during correct vs. wrong trials ($\Delta PLV_{\theta} = -0.387 \pm 0.059$, $p < 10^{-6}$, $\Delta PLV_{\alpha} = -0.331 \pm 0.073$, $p < 10^{-4}$, $n = 92$ sites).

Within IT, the PLV in the theta and alpha bands increased during the visual period compared to baseline ($\Delta PLV_{\theta} = 1.158 \pm 0.143$, $p < 10^{-9}$, $\Delta PLV_{\alpha} = 1.343 \pm 0.224$, $p < 10^{-6}$, $n = 69$ sites). PLV was not object selective in any frequency band during the visual period. During the delay period, PLV in the theta and alpha bands decreased compared to baseline ($\Delta PLV_{\theta} = -0.470 \pm 0.083$, $p < 10^{-6}$, $\Delta PLV_{\alpha} = -0.203 \pm 0.081$, $p = 0.015$, $n = 69$ sites). PLV was not object selective in any frequency band during the delay period. Theta band PLV was lower on correct trials compared to wrong trials ($\Delta PLV_{\theta} = -0.206 \pm 0.071$, $p = 0.007$, $n = 68$ sites).

LFP power-power correlations between areas:

In order to control for whether the PPL results reflect the correlation of activity between areas, we calculated power-power correlation between areas and their relationship to performance. There was no difference in LFP power correlations between FEF and IT for correct vs. wrong trials in any frequency band, and beta band power correlations did not show object or location selectivity (full statistics in Table S1).

Supplementary Figures

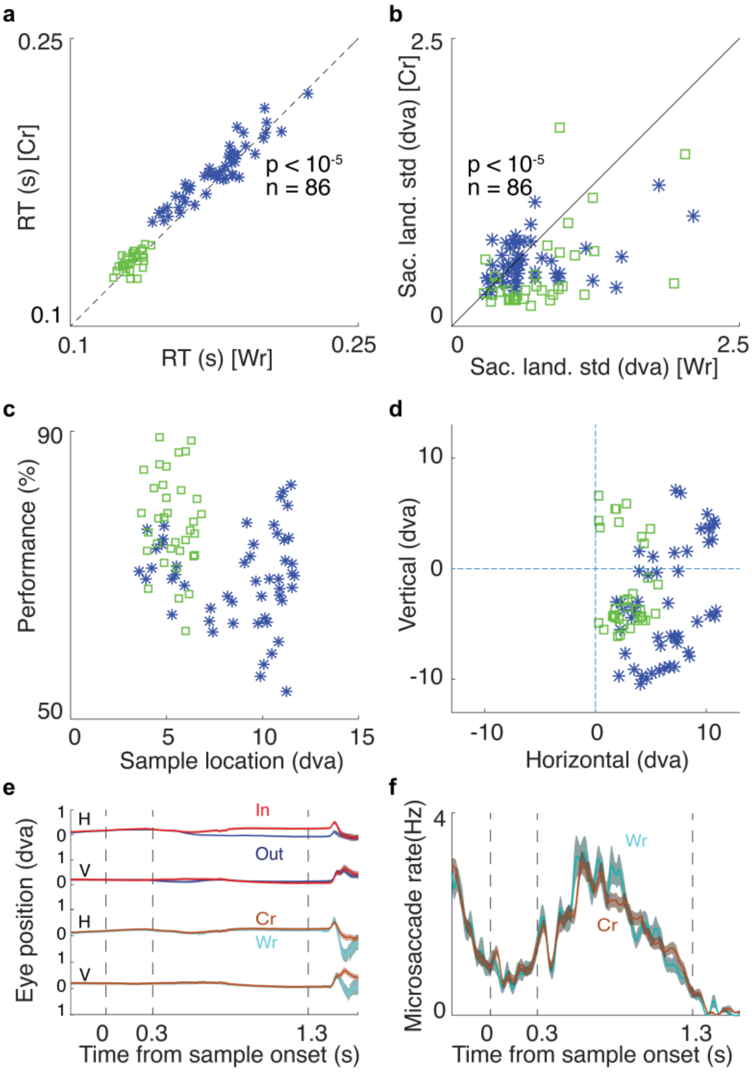

Figure S1. Behavioral performance. For (a-d), data shown separately for M1 (blue) and M2 (green). a, Reaction time for correct vs. wrong trials across sessions. Each point represents the median reaction times for correct and wrong trials in one experimental session. Correct responses were slower than wrong responses ($p < 10^{-5}$, n = 86 sessions, Wilcoxon's signed-rank test, two-sided). M1 performed the task at a greater average eccentricity and had lower average performance than M2. b, Variability in saccade landing points for correct vs. wrong trials across sessions. Landing point variability was greater on wrong trials ($p < 10^{-5}$, n = 86 sessions, Wilcoxon's signed-rank test, two-sided). Each point represents the average of the standard deviations of saccade landing points for the two target positions in a single session. c, Performance as a function of sample eccentricity; averaged across all trials for each session. d, Estimated FEF RF position for all sessions. For the In condition, the sample was placed at the RF center. e, Eye position during the task, in the (V) vertical and (H) Horizontal plane, for In (red) vs. Out (blue) and correct (brown) and wrong (cyan) trials. Data represented as mean ± SEM. In (a-e) 6 sessions were excluded because of a lack of eye data. f, Microsaccade rate over the timecourse of a trial for correct (brown) and wrong (cyan) trials, shown as mean ± SEM (n = 86 sessions).

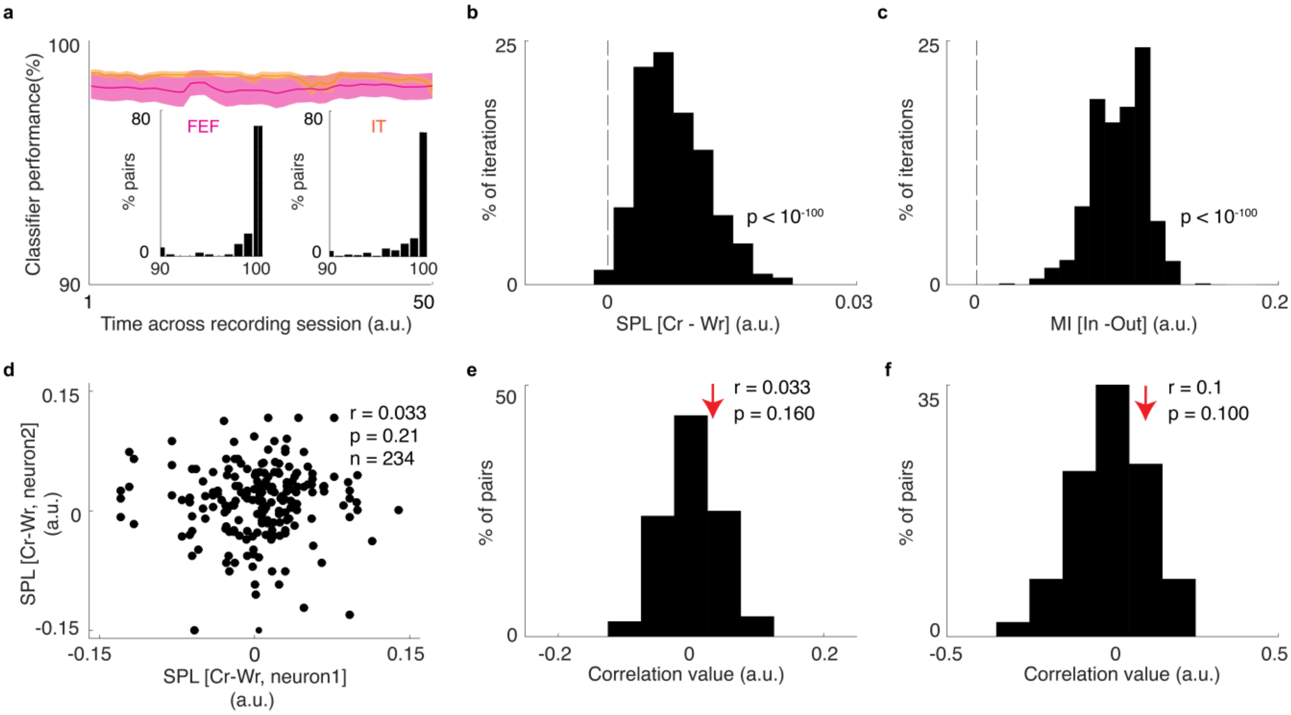

Figure S2. Evaluation of the quality of collected data. a, SVM classifier performance on waveform clusters is high. Plot shows the average classifier performance (mean ± SEM) in categorizing the spike waveforms of simultaneously recorded neurons as a function of time across the recording session (spikes across each session were split into 50 bins) for FEF (pink, n =105 waveform pairs) and IT (orange, n = 301 waveform pairs). Inset histograms show the distribution of average classifier performance across cluster pairs for each session, in FEF (left) and IT (right). b, Control for Fig. 4a with one unit per recording session. Histogram shows the distribution of the median difference in correct vs. wrong SPL for 1000 subsamples (each with 79 spike-LFP pairs, a maximum of one unit per recording session); the median of this distribution was significantly greater than zero ($\Delta SPL_{Cr\text{-}Wr} = 0.008 \pm 0.000$, $p < 10^{-10}$, n = 1000 subsamples, Wilcoxon's signed-rank test, two-sided). c, Control for Fig. 5e with one unit per recording session. Histogram shows the distribution of the median difference in modulation index (modulation of object discriminability for High vs. Low PPL trials) for In vs. Out, for 1000 subsamples (each with 40 units, a maximum of one unit per recording session); the median of this distribution was significantly greater than zero ($\Delta MI_{In\text{-}Out} = 0.090 \pm 0.000$, $p < 10^{-10}$, n = 1000 subsamples, Wilcoxon's signed-rank test, two-sided). d, Control for figure 4a: scatterplot shows the correlation between SPL effect size (correct – wrong) for pairs of neurons recorded from same electrode (Kendall correlation, r = 0.033, p = 0.210, n = 234 pairs). e, Control for figure 4a: Histogram shows the distribution of correlation values for 100 subsamples with n = 234 pairs each, as in (d) of randomly selected pairs of neurons from different electrodes. Red arrow shows the correlation value for same-electrode pairs, not significantly different from the median of the distribution (p = 0.160, n = 100 subsamples, Wilcoxon's signed-rank test, two-sided). f, Control for figure 5e: similar to (e) but for the MI effect (In – Out); there was no significant difference between the correlation coefficient for same-electrode pairs and different-electrode pairs (p = 0.100, n = 100 subsamples, Wilcoxon's signed-rank test, two-sided).

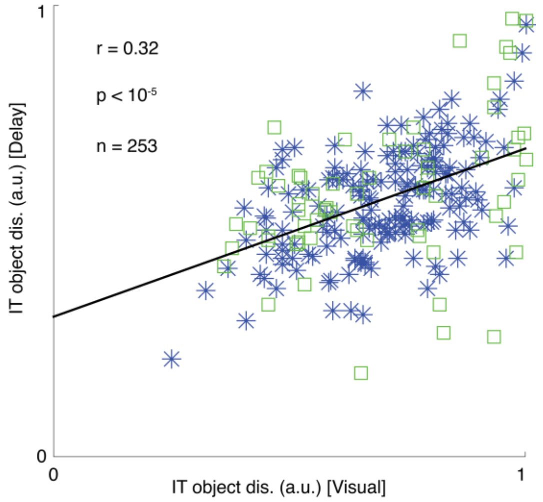

Figure S3. Object discriminability during the delay and visual periods was correlated. Object discriminability during the task, for M1 (blue) and M2 (green), was correlated between the visual and delay periods (Kendall correlation, r = 0.32, $p < 10^{-5}$, n = 253 IT units). Each point shows the ability of one IT unit to discriminate between two objects (measured with ROC).

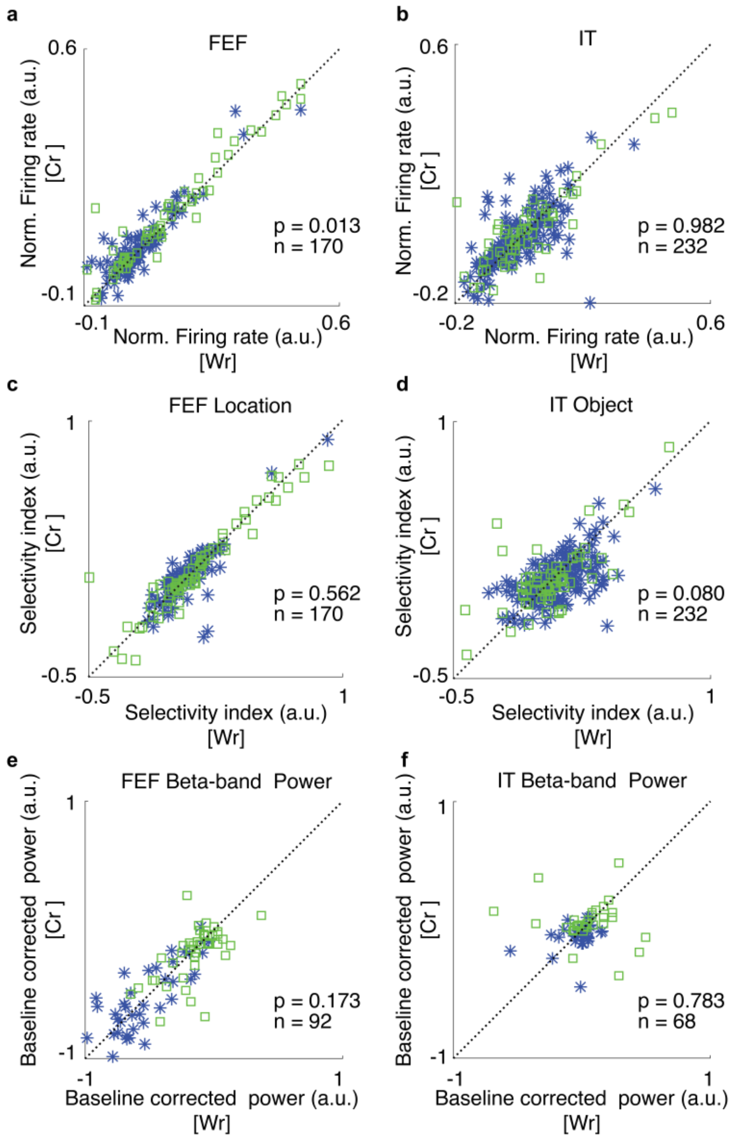

Figure S4. Behavioral correlations of firing rate, selectivity, and beta LFP power in FEF and IT. Scatter plots show firing rate (a-b, n = 170 FEF units, n = 232 IT units), selectivity index for object information (c, n = 170 FEF units) or location information (d, n = 232 IT units), and beta band LFP power (e-f, n = 92 FEF sites, n = 68 IT sites), for correct vs. wrong trials, for FEF (a,c,e) and IT (b,d,f). M1 plotted in blue and M2 in green. All p-values from two-sided Wilcoxon's signed-rank tests.

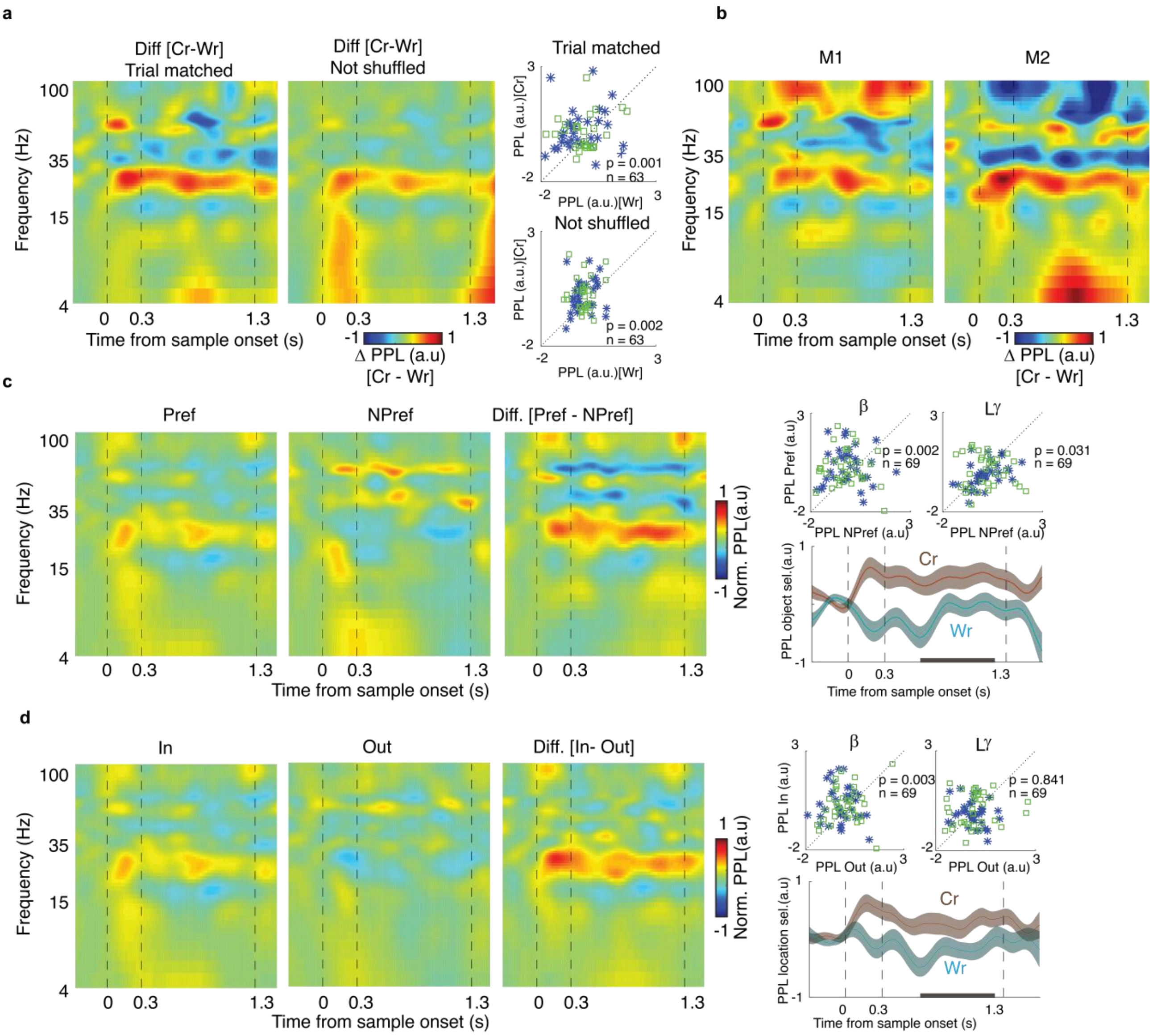

Figure S5. PPL trial matching, M1 vs. M2, object and location coding. a, Beta band PPL was greater for correct trials when trial numbers were matched, and without the shuffle correction. Heatmaps show the time-frequency map of PPL, normalized to baseline across the course of the DMS task (n = 63 LFP recordings), when the number of correct and wrong trials were matched (left) and without shuffle correction (right). Scatterplots illustrate the average beta PPL for the correct vs. wrong trials with trial matching (top) and without shuffle correction (bottom); p-values from two-sided Wilcoxon's signed-rank tests. b, The trend toward higher beta band PPL on correct trials was present in both M1 and M2. Heatmaps show the time-frequency map of PPL difference between correct and wrong trials, across the course of the DMS task for the IN condition in M1 (left; n = 34 LFP recordings) and M2 (right; n = 29 LFP recordings). c, Inter-areal beta PPL encoded object identity during the delay period. Heatmap shows the time-frequency map of PPL, normalized to baseline across the course of the DMS task (n = 69 LFP pairs; correct trials, IN condition) for Pref (left) and NPref (middle) trials, and the difference between them (right). Scatter plots in the upper right show the mean delay-period PPL in the beta and

low-gamma frequency bands for Npref (x-axis) vs. Pref (y-axis); p-values from two-sided Wilcoxon's signed-rank tests. Bottom right: Timecourse of object selectivity for correct (brown) and wrong (cyan) trials, mean ± SEM. d, Inter-areal beta PPL encoded the location of the sample during the delay period. Heatmap shows the time-frequency map of PPL, normalized to baseline across the course of the DMS task (n = 69 LFP pairs; correct trials, Pref condition) for In (left), Out (middle), and the difference between them (right). Scatter plots in the upper right show the mean delay-period PPL in the beta and low-gamma frequency bands for Out (x-axis) vs. In (y-axis); p-values from two-sided Wilcoxon's signed-rank test. Bottom right: Timecourse of location selectivity for correct (brown) and wrong (cyan) trials, mean ± SEM. All scatterplots show M1 in blue and M2 in green.

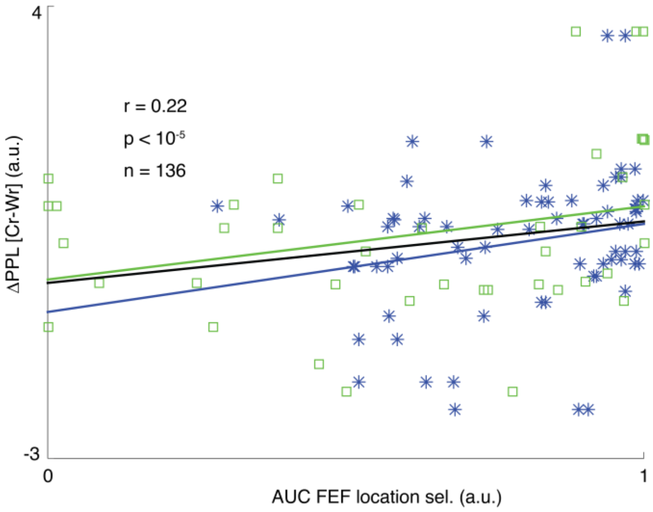

Figure S6. Relationship between the magnitude of FEF spatial selectivity and the change in FEF-IT PPL with performance. Scatter plot shows the magnitude of FEF spatial selectivity (area under the curve, ROC In vs. Out) for each FEF unit (x-axis) and the difference in PPL value at that site for correct vs. wrong trials (y-axis). There is a positive correlation between the strength of FEF spatial selectivity and the change in PPL for correct vs. wrong trials (Kendall correlation, r = 0.22, $p < 10^{-5}$, n = 136 FEF units). Scatterplots show M1 in blue and M2 in green, and lines show the fit for each monkey (blue, green) and combined date (black).

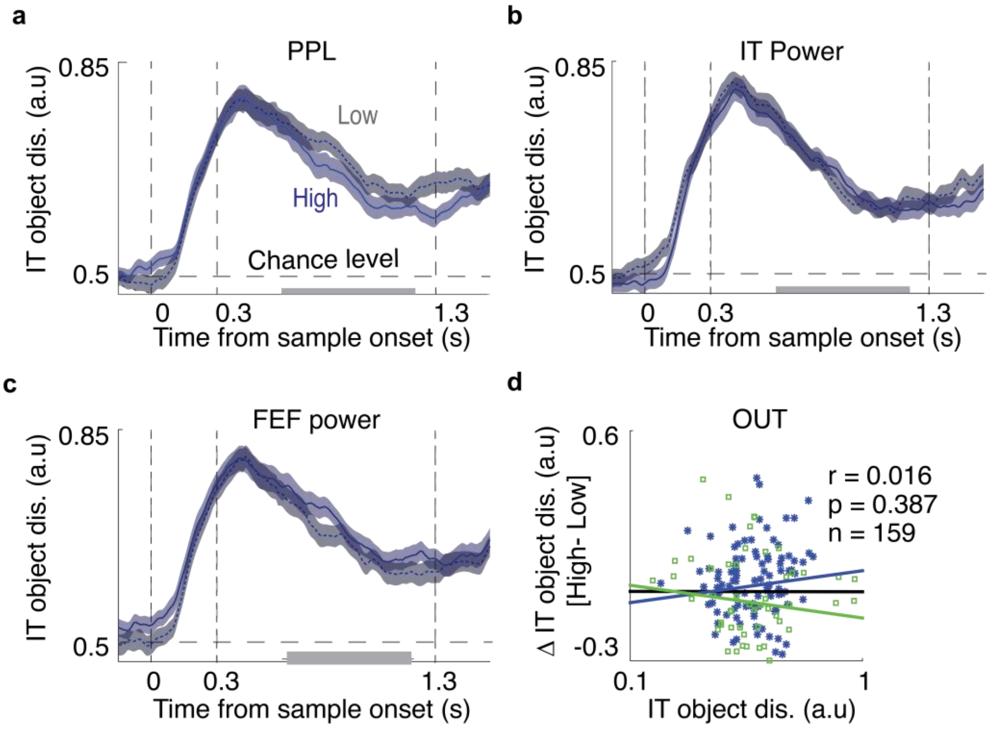

Figure S7. Object coding in IT firing rate during the Out condition. Similar to Figure 5, for the Out rather than the In condition. a, Timecourse of the object discriminability values for High (blue) vs. Low (grey) PPL trials for object-selective IT units (n = 73 units), for the Out condition. Data were smoothed within a window of 1ms and represented as mean ± SEM. Gray bar indicates portion of delay period used for analysis in (d). b-c, Same as (a) but with trials split according to High or Low beta-band IT (b, n = 74 units) or FEF (c, n = 71 units) LFP power. d, Change in IT object discriminability (High vs. Low PPL trials) as a function of discriminability, n = 159 units, Kendall correlation, r = 0.016, p = 0.387). Lines show fits for individual monkeys (blue, green) and combined data (black).

Statistics	M1	M2	Combined	Effect size
Figure S1a<br>Reaction times<br>(Cr vs. Wr)	$p < 10^{-4}$<br>Δ =4.18±0.87<br>n = 51	$p < 10^{-3}$<br>Δ = 2.63 ±0.63<br>n = 35	$p < 10^{-6}$<br>Δ =3.55±0.58<br>n = 86	6.091
Figure S1b<br>Saccade landing variability<br>(Cr vs. Wr)	p =0.004<br>Δ =-0.15±0.05<br>n = 51	$p < 10^{-4}$<br>Δ = -0.27 ±0.05<br>n = 35	$p < 10^{-6}$<br>Δ =-0.20±0.03<br>n = 86	5.823
Figure 1b, S4a<br>Firing rate of FEF<br>(Cr vs. Wr)	p = 0.122<br>Δ = 0.006±0.005<br>n = 102	p = 0.031<br>Δ =0.011±0.005<br>n = 68	p = 0.013<br>Δ = 0.008±0.003<br>n = 170	2.338
Figure 1b, S4b<br>Firing rate of IT<br>(Cr vs. Wr)	p = 0.791<br>Δ = -0.001±0.006<br>n = 171	p = 0.623<br>Δ =-0.002±0.009<br>n = 61	p = 0.982<br>Δ = -0.002±0.005<br>n = 232	0.325
Figure S4c<br>Location selectivity in firing rate of FEF<br>(Cr vs. Wr)	p = 0.204<br>Δ =0.000± 0.008<br>n = 102	p = 0.537<br>Δ =-0.001± 0.012<br>n = 68	p = 0.562<br>Δ =0.000± 0.007<br>n = 170	0.072
Figure S4d<br>Object selectivity in firing rate of IT<br>(Cr vs. Wr)	p = 0.031<br>Δ =-0.026±0.010<br>n = 171	p = 0.872<br>Δ = 0.014±0.020<br>n = 61	p = 0.080<br>Δ =-0.016±0.009<br>n = 232	1.699
Figure 1d, S4e<br>Beta band LFP power of FEF<br>(Cr vs. Wr)	p = 0.298<br>Δ =-0.017± 0.044<br>n = 51	p = 0.389<br>Δ =-0.034± 0.028<br>n = 41	p = 0.173<br>Δ =-0.024± 0.027<br>n = 92	0.902
Figure 1d, S4f<br>Beta band LFP power of IT<br>(Cr vs. Wr)	p = 0.646<br>Δ = 0.016±0.036<br>n = 35	p = 0.426<br>Δ = 0.063±0.071<br>n = 33	p = 0.783<br>Δ = 0.039±0.039<br>n = 68	0.990
Figure 2a-c<br>Beta band PPL<br>(Cr vs. Wr)	p = 0.009<br>Δ =0.468±0.183<br>n = 34	p = 0.034<br>Δ =0.475± 0.201<br>n = 29	p <0.001<br>Δ = 0.471±0.134<br>n = 63	3.513
Figure S5c<br>Beta band PPL Object<br>(Pref vs. NPref)	p = 0.049<br>Δ =0.411±0.214<br>n = 35	p = 0.018<br>Δ =0.556± 0.248<br>n = 34	p =0.002<br>Δ = 0.482±0.163<br>n = 63	2.965
Figure S5d<br>Beta band PPL Location<br>(In vs. Out)	p = 0.023<br>Δ =0.495±0.203<br>n = 35	p = 0.064<br>Δ =0.290± 0.199<br>n = 34	p =0.003<br>Δ = 0.394±0.142<br>n = 69	2.778
Figure 2d<br>Beta band PPL Object sel. (Cr vs. Wr)	p = 0.102<br>Δ =0.507±0.249<br>n = 34	p = 0.005<br>Δ =0.825± 0.235<br>n = 29	p =0.002<br>Δ = 0.654±0.173<br>n = 63	3.783
Figure 2e<br>Beta band PPL Location sel. (Cr vs. Wr)	p = 0.006<br>Δ =0.776±0.259<br>n = 34	p = 0.021<br>Δ =0.458± 0.191<br>n = 29	p <0.001<br>Δ = 0.633±0.166<br>n = 63	3.815
Figure S5b<br>Theta band PPL<br>(Cr vs. Wr)	p = 0.059<br>Δ =-0.248±0.126<br>n = 34	p = 0.029<br>Δ =0.473± 0.196<br>n = 29	p =0.733<br>Δ = 0.083±0.121<br>n = 63	0.691
Figure 4a<br>SPL FEF phase -IT Spike<br>(Cr vs. Wr)	p = 0.014<br>Δ =0.006±0.004<br>n = 168	p = 0.016<br>Δ =0.011± 0.008<br>n = 53	p <0.001<br>Δ = 0.007±0.003<br>n = 221	2.134
Figure 4b<br>SPL IT phase -IT Spike<br>(Cr vs. Wr)	p = 0.315<br>Δ =0.003±0.004<br>n = 125	p = 0.131<br>Δ =0.012± 0.005<br>n = 57	p =0.088<br>Δ = 0.006±0.003<br>n = 182	1.805
Figure 4c<br>SPL FEF phase -FEF Spike<br>(Cr vs. Wr)	p = 0.927<br>Δ =-0.001±0.002<br>n = 102	p = 0.415<br>Δ =-0.003± 0.004<br>n = 68	p =0.570<br>Δ = -0.002±0.002<br>n = 170	0.471
Figure 4d<br>SPL IT phase -FEF Spike<br>(Cr vs. Wr)	p = 0.181<br>Δ =0.007±0.006<br>n = 69	p = 0.574<br>Δ =0.004± 0.005<br>n = 51	p =0.167<br>Δ = 0.006±0.004<br>n = 120	1.435
Figure 5e<br>Object selectivity<br>(In vs. Out)	p = 0.016<br>Δ =0.051±0.026<br>n = 50	p = 0.002<br>Δ =0.127± 0.033<br>n = 23	p <0.001<br>Δ = 0.075±0.021<br>n = 73	3.589
Figure 5f<br>Correlation between IT unit<br>discriminability and Δ High vs. Low (In)	r=0.100<br>p =0.077<br>n = 102	r=0.086<br>p =0.172<br>n = 57	r=0.100<br>p =0.024<br>n = 159	-
Figure S7d<br>Correlation between IT unit<br>discriminability and Δ High vs. Low (Out)	r=0.081<br>p =0.108<br>n = 103	r=-0.082<br>p =0.178<br>n = 56	r=0.015<br>p =0.390<br>n = 159	-
Figure S5d<br>Correlation between FEF spatial<br>selectivity and Δ Cr vs.Wr PPL	r=0.229<br>p =0.008<br>n = 77	r=0.284<br>p =0.055<br>n = 59	r=0.220<br>$p < 10^{-5}$<br>n = 136	-

Table S1. Statistics for individual monkeys. Left column indicates the measure and figures in which results are plotted. Columns show difference (Δ, mean ± SEM), significance (p), and sample size (n) for each monkey individually, and combined. Statistical comparisons use a two -sided Wilcoxon's

signed-rank test; correlations use the Kendall correlation. Last column shows effect size (Cohen's d, see Methods) for each measurement, reflecting the difference between the means; effect sizes >2 are considered a "huge effect" [5]. Note that the majority of our effects, most importantly the key findings (Fig. 2a-c, 4a, 5e), show significance of $p<0.001$ and so would still be significant with Bonferroni correction of at least n=50 multiple comparisons. Green boxes indicate statistical significance.

		IT		*FEF*		*FEF- IT Interaction*		
		Performance	Object	Performance	Location	Performance	Object	Location
Firing rate		p = 0.982<br>Δ = -0.002± 0.005<br>n = 232	$p < 10^{-15}$<br>Δ =0.046± 0.005<br>n = 235	p =0.013<br>Δ = 0.008±0.003<br>n = 170	$p < 10^{-8}$<br>Δ =0.059±0.011<br>n = 170			
LFP power	θ	p = 0.396<br>Δ = -0.069± 0.051<br>n = 68	p = 0.453<br>Δ =-0.030±0.047<br>n = 69	p = 0.160<br>Δ =-0.037±0.030<br>n = 92	$p < 10^{-6}$<br>Δ =-0.192±0.032<br>n = 92	p = 0.059<br>Δ =-0.194±0.101<br>n = 63	p = 0.035<br>Δ =-0.282±0.124<br>n = 69	p = 0.172<br>Δ =-0.133±0.102<br>n = 69
	α	p = 0.310<br>Δ = -0.017± 0.033<br>n = 68	p = 0.998<br>Δ =-0.024±0.030<br>n = 69	p = 0.277<br>Δ = 0.007±0.026<br>n = 92	$p < 10^{-6}$<br>Δ =-0.024±0.032<br>n = 92	p = 0.556<br>Δ =-0.061±0.108<br>n = 63	p = 0.149<br>Δ =-0.133±0.094<br>n = 69	p = 0.185<br>Δ =-0.050±0.113<br>n = 69
	β	p = 0.783<br>Δ =0.039±0.039<br>n = 68	p = 0.486<br>Δ =-0.009±0.026<br>n = 69	p = 0.173<br>Δ =-0.024±0.027<br>n = 92	$p < 10^{-4}$<br>Δ =0.086± 0.030<br>n = 92	p = 0.753<br>Δ = 0.075±0.133<br>n = 63	p = 0.170<br>Δ =0.207±0.124<br>n = 69	p = 0.560<br>Δ = 0.047±0.129<br>n = 69
	Lγ	p = 0.285<br>Δ =0.006±0.0160<br>n = 68	p = 0.439<br>Δ = 0.004±0.007<br>n = 69	p = 0.061<br>Δ =-0.029±0.031<br>n = 92	$p < 10^{-4}$<br>Δ = 0.115±0.028<br>n = 92	p = 0.074<br>Δ =-0.292±0.130<br>n = 63	p = 0.556<br>Δ = 0.118±0.117<br>n = 69	p = 0.025<br>Δ = 0.271±0.121<br>n = 69
	Hγ	p = 0.112<br>Δ =0.002±0.008<br>n = 68	p = 0.013<br>Δ = 0.011±0.006<br>n = 69	p = 0.104<br>Δ =-0.018±0.030<br>n = 92	$p < 10^{-8}$<br>Δ = 0.241±0.056<br>n = 92	p = 0.419<br>Δ =-0.057±0.120<br>n = 63	p = 0.767<br>Δ =-0.015±0.106<br>n = 69	p = 0.888<br>Δ =-0.019±0.113<br>n = 69
LFP phase	*θ*	p = 0.007<br>Δ =-0.206±0.071<br>n = 68	p = 0.163<br>Δ =-0.059±0.061<br>n = 69	$p < 10^{-7}$<br>Δ =-0.386±0.059<br>n = 92	$p < 10^{-11}$<br>Δ =-0.828±0.088<br>n = 92	p = 0.733<br>Δ =0.083± 0.121<br>n = 63	p = 0.548<br>Δ =0.001± 0.116<br>n = 69	p = 0.795<br>Δ =0.072± 0.096<br>n = 69
	α	p = 0.116<br>Δ =-0.149±0.086<br>n = 68	p = 0.311<br>Δ = 0.078±0.087<br>n = 69	$p < 10^{-6}$<br>Δ =-0.332±0.073<br>n = 92	p = 0.001<br>Δ =-0.268±0.087<br>n = 92	p = 0.267<br>Δ =0.083± 0.101<br>n = 63	p = 0.717<br>Δ =0.050± 0.079<br>n = 69	p=0.776<br>Δ = 0.041±0.072<br>n = 69
	β	p = 0.208<br>Δ =-0.132±0.131<br>n = 68	p = 0.686<br>Δ =-0.062±0.137<br>n = 69	P = 0.714<br>Δ =-0.035±0.090<br>n = 92	p = 0.429<br>Δ =0.110± 0.121<br>n = 92	p < 0.001<br>Δ = 0.471±0.134<br>n = 63	p = 0.002<br>Δ = 0.483±0.163<br>n = 69	p = 0.003<br>Δ = 0.394±0.142<br>n = 69
	Lγ	p = 0.165<br>Δ =- 0.146± 0.095<br>n = 68	p = 0.754<br>Δ = -<br>0.054±0.116<br>n = 69	p = 0.809<br>Δ = 0.002±0.091<br>n = 92	p = 0.901<br>Δ =-0.019±0.124<br>n = 92	p = 0.249<br>Δ =-0.153±0.140<br>n = 63	p = 0.031<br>Δ =-0.219±0.122<br>n = 69	p = 0.841<br>Δ = 0.011±0.132<br>n = 69
	Hγ	p = 0.399<br>Δ = 0.024± 0.064<br>n = 68	p = 0.983<br>Δ = 0.007±0.084<br>n = 69	p = 0.203<br>Δ =-0.067±0.058<br>n = 92	p = 0.246<br>Δ =-0.101±0.072<br>n = 92	p = 0.091<br>Δ =-0.138±0.096<br>n = 63	p = 0.262<br>Δ =-0.155±0.088<br>n = 69	p = 0.584<br>Δ =-0.073±0.101<br>n = 69

Table S2. Statistics for relationship between within-area and inter-areal measures and object selectivity, location selectivity, and performance. Neural measure is indicated by the label on the left (firing rate, LFP power or phase in a specific frequency band). Area (FEF, IT, or inter-areal) is listed at the top. Selectivity measure (performance, Cr vs. Wr; location, In vs. Out; object, Pref vs. NPref) is indicated in the second row above the relevant column. Performance measures were calculated based on the Pref condition for IT, on the In condition for FEF and on the Pref, In condition for the interactions. Each box indicates the magnitude of the difference between conditions (Δ, mean ± SEM), significance (p), and sample size (n) for the corresponding neural and selectivity measure. Statistical comparisons use a two-sided Wilcoxon's signed-rank test. Color indicates significant increases (green) and decreases (gray) for the Cr, In, or Pref condition.

References:

1. Lowet, E. *et al.* Enhanced neural processing by covert attention only during microsaccades directed toward the attended stimulus. *Neuron* **99**, 207–214 (2018).
2. Weyand, T. G. & Malpeli, J. G. Responses of neurons in primary visual cortex are modulated by eye position. *J. Neurophysiol.* **69**, 2258–2260 (1993).
3. Martinez-Conde, S., Otero-Millan, J. & Macknik, S. L. The impact of microsaccades on vision: towards a unified theory of saccadic function. *Nat. Rev. Neurosci.* **14**, 83–96 (2013).
4. Liebe, S., Hoerzer, G. M., Logothetis, N. K. & Rainer, G. Theta coupling between V4 and prefrontal cortex predicts visual short-term memory performance. *Nat. Neurosci.* **15**, 456–462 (2012).
5. Sawilowsky, S. S. New effect size rules of thumb. *J. Mod. Appl. Stat. Methods* **8**, 26 (2009).
