# Supplementary material for: Frontotemporal coordination predicts working memory performance and its local neural signatures
Source: Nat Commun. 2021 Feb 17;12:1103. doi: 10.1038/s41467-021-21151-1 (PMC7889930; doi:10.1038/s41467-021-21151-1)
Supplement: Supplementary file 3 — Reporting Summary [file 41467_2021_21151_MOESM3_ESM.pdf]

nature research

Corresponding author(s): Behrad Noudoost

Last updated by author(s): Jan 23, 2020

Reporting Summary

Nature Research wishes to improve the reproducibility of the work that we publish. This form provides structure for consistency and transparency in reporting. For further information on Nature Research policies, see Authors & Referees and the Editorial Policy Checklist.

Statistics

For all statistical analyses, confirm that the following items are present in the figure legend, table legend, main text, or Methods section.

n/a	Confirmed	
☐	☒	The exact sample size (*n*) for each experimental group/condition, given as a discrete number and unit of measurement
☐	☒	A statement on whether measurements were taken from distinct samples or whether the same sample was measured repeatedly
☐	☒	The statistical test(s) used AND whether they are one- or two-sided<br>*Only common tests should be described solely by name; describe more complex techniques in the Methods section.*
☐	☒	A description of all covariates tested
☐	☒	A description of any assumptions or corrections, such as tests of normality and adjustment for multiple comparisons
☐	☒	A full description of the statistical parameters including central tendency (e.g. means) or other basic estimates (e.g. regression coefficient) AND variation (e.g. standard deviation) or associated estimates of uncertainty (e.g. confidence intervals)
☐	☒	For null hypothesis testing, the test statistic (e.g. *F*, *t*, *r*) with confidence intervals, effect sizes, degrees of freedom and *P* value noted<br>*Give P values as exact values whenever suitable.*
☒	☐	For Bayesian analysis, information on the choice of priors and Markov chain Monte Carlo settings
☒	☐	For hierarchical and complex designs, identification of the appropriate level for tests and full reporting of outcomes
☐	☒	Estimates of effect sizes (e.g. Cohen's *d*, Pearson's *r*), indicating how they were calculated

Our web collection on statistics for biologists contains articles on many of the points above.

Software and code

Policy information about availability of computer code

Data collection	Stimulus presentation and juice delivery were controlled using custom software, written in MATLAB 2015a using the MonkeyLogic toolbox for monkey 1 experiments. Stimulus presentation, data acquisition and behavioral monitoring were controlled by the CORTEX system for monkey 2 experiments.
Data analysis	All analyses were carried out with custom code written in Matlab 2015a (MathWorks) using the Signal processing toolbox.

For manuscripts utilizing custom algorithms or software that are central to the research but not yet described in published literature, software must be made available to editors/reviewers. We strongly encourage code deposition in a community repository (e.g. GitHub). See the Nature Research guidelines for submitting code & software for further information.

Data

Policy information about availability of data

All manuscripts must include a data availability statement. This statement should provide the following information, where applicable:

- Accession codes, unique identifiers, or web links for publicly available datasets
- A list of figures that have associated raw data
- A description of any restrictions on data availability

The datasets generated during the current study are available from the corresponding author on reasonable request.

Field-specific reporting

Please select the one below that is the best fit for your research. If you are not sure, read the appropriate sections before making your selection.

☒ Life sciences ☐ Behavioural & social sciences ☐ Ecological, evolutionary & environmental sciences

For a reference copy of the document with all sections, see nature.com/documents/nr-reporting-summary-flat.pdf

Life sciences study design

All studies must disclose on these points even when the disclosure is negative.

Sample size	Data are reported from 58 IT sites (36 M1, 22 M2) and 86 FEF sites (51 M1, 35 M2). SPL measurements in figure 3 were performed on 209 spike-LFP pairs (IT units, FEF LFP), 96 spike-LFP pairs (FEF units, IT LFP), and 140 spike-LFP pairs (IT units, IT LFP) in both monkeys. Data collection was based on numbers of units typically required to reach significance in similar neurophysiological studies.
Data exclusions	LFP data of 28 IT sites (14 M1, 14 M2) was discarded prior to any analysis due to noise or data acquisition issues. As is standard practice, when comparing correct and wrong trials, certain sites and units were excluded due to low numbers of wrong trials (3 wrong trials, Lundqvist et al 2018); number of sites and units included in each analysis are included in the text with the statistics. As is typical in analysis of visual selectivity, sites without selective responses were excluded (Land et al 2013): for IT LFPs, object selectivity of the site was determined based on local multiunit responses; in figure 2d and figure S1c, LFPs from 7 IT sites were excluded from analysis because multiunit activity was not object selective.
Replication	Effects were successful in two animals, which is the standard in non-human primate models.
Randomization	In the behavioral task, sample location, sample identity, matching target location, and nonmatching target identity was selected randomly without replacement within a set of trials consisting of all possible combinations of two sample locations, three potential sample identities, two target locations, and two nonmatching target identities.
Blinding	N/A: all analysis of neural data was performed by code applied to all conditions.

Reporting for specific materials, systems and methods

We require information from authors about some types of materials, experimental systems and methods used in many studies. Here, indicate whether each material, system or method listed is relevant to your study. If you are not sure if a list item applies to your research, read the appropriate section before selecting a response.

Materials & experimental systems

n/a	Involved in the study	
☒	☐	Antibodies
☒	☐	Eukaryotic cell lines
☒	☐	Palaeontology
☐	☒	Animals and other organisms
☒	☐	Human research participants
☒	☐	Clinical data

Methods

n/a	Involved in the study	
☒	☐	ChIP-seq
☒	☐	Flow cytometry
☒	☐	MRI-based neuroimaging

Animals and other organisms

Policy information about studies involving animals; ARRIVE guidelines recommended for reporting animal research

Laboratory animals	2 adult male rhesus monkeys (Macaca mulatta, 10 and 11 kg, 11 and 13 years old) were used in this study
Wild animals	The study did not involve wild animals.
Field-collected samples	The study did not involve field-collected samples.
Ethics oversight	All experimental procedures were in accordance with the National Institutes of Health Guide for the Care and Use of Laboratory Animals and the Society for Neuroscience Guidelines and Policies. The protocols for all experimental, surgical, and behavioral procedures were approved by the Institute Fundamental Science committee for monkey 1, and by the Stanford University Institutional Animal Care and Use Committee for monkey 2.

Note that full information on the approval of the study protocol must also be provided in the manuscript.
